# Airway foreign body manifested as a coin lesion

**DOI:** 10.1002/ccr3.1754

**Published:** 2018-08-08

**Authors:** Yuko Nishinaga, Shinichi Miyazaki, Ryo Yamashita, Takuya Ikeda

**Affiliations:** ^1^ Department of Respiratory Medicine Nagoya University Graduate School of Medicine Nagoya Japan; ^2^ Department of Respiratory Medicine Yokkaichi Municipal Hospital Yokkaichi‐shi Japan

**Keywords:** aspiration, cough, foreign body

## Abstract

Foreign body aspiration is a potentially life‐threatening event. The nature of the inhaled objects is highly variable, ranging from organic to inorganic material. Although most pills are radiolucent, lanthanum carbonate is radiopaque and may be identified on chest X‐rays.

## 1 CASE DESCRIPTION

**Figure 1:**
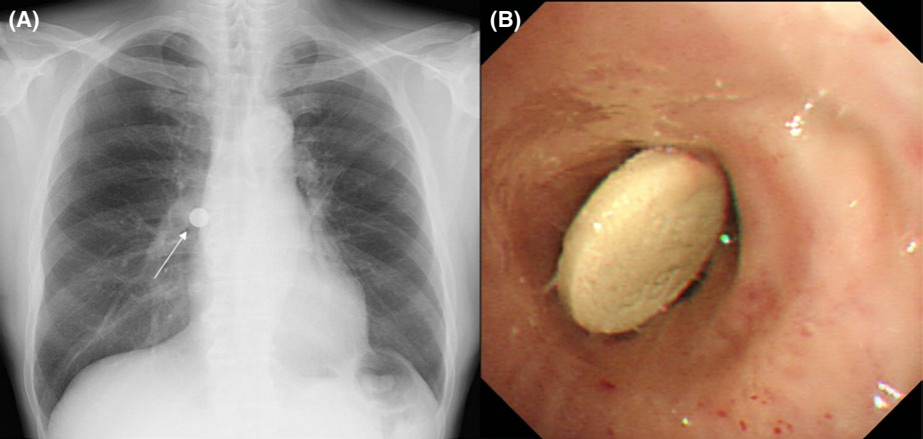
(A) Chest radiograph on admission, (B) bronchoscopic image showing foreign body in right bronchus intermedius.

A 58‐year‐old nonsmoking male was referred with a 1‐week history of cough. He had been on peritoneal dialysis since 3 years due to hypertensive nephrosclerosis. Despite having sustained an intracranial hemorrhage 8 years prior, he had no residual neurologic deficits. A chest radiograph revealed a radiopaque foreign body in the right hilar region (Figure 1A). The presence of a bronchial foreign body was suspected, and flexible bronchoscopy was performed. Balloon catheter was dilated at the periphery of the foreign body and withdrawn together with the bronchoscope (Figure 1B). After confirming the history of the prescribed medicines, the removed pill was found to be of lanthanum carbonate. After the procedure, the patient's respiratory symptoms resolved.

The nature of airway foreign bodies is highly variable, ranging from organic to inorganic. It is estimated that approximately 7% of all foreign bodies are medicinal pills.^1^ Although most pills are radiolucent, lanthanum carbonate is radiopaque and may be identified on chest X‐rays. Because the removed pill was a chewable tablet, it was not crushed during the procedure.

## CONFLICT OF INTEREST

None declared.

## AUTHORSHIP

YN: reviewed the patient, performed the literature review, and wrote the manuscript. SM: involved in critical revision of the work. RY and TI: supervised the project.

## INFORMED CONSENT

We obtained the patient's informed consent to conduct this study.
